# Higher-level phylogeny of Chrysomelidae based on expanded sampling of mitogenomes

**DOI:** 10.1371/journal.pone.0258587

**Published:** 2022-01-21

**Authors:** Heng Zhang, Nan Song, Xinming Yin

**Affiliations:** College of Plant Protection, Henan Agricultural University, Zhengzhou, China; Sichuan University, CHINA

## Abstract

Chrysomelidae is one of the most diverse lineages of beetles. The classification and phylogeny of Chrysomelidae have been contentious. In this study, we obtained 16 new mitogenome sequences by using next-generation sequencing. Combined with the published mitogenomes, we inferred the phylogenetic relationships of Chrysomelidae. Different data recoding strategies and substitution models were applied to phylogenetic reconstruction. In the Maximum likelihood analyses under the homogeneous model, Dayhoff recoding allowed for the improved phylogenetic resolution due to the decreased level of heterogeneous sequence divergence. Bayesian inference under the heterogeneous model yielded generally well resolved subfamily relationships. The present mitogenome data strongly supported Chrysomelidae as a monophyletic group. Consistent with previous work, we found three major subfamily clades within Chrysomelidae. However, the pattern of the “sagrine” clade plus the “eumolpine” clade being sister to the “chrysomeline” clade contrasted with the prior study. The placement of the genus *Syneta* with regards to these three clades was ambiguous. Relationships recovered suggested several major chrysomelid lineages, including: (1) Bruchinae in the “sagrine” clade; (2) Donaciinae + Criocerinae; (3) Spilopyrinae + (Cassidinae + (Eumolpinae + (Lamprosomatinae + Cryptocephalinae))); (4) Chrysomelinae + (Alticinae + Galerucinae). Results also suggested the placement of *Timarcha* outside the major Chrysomelinae.

## Introduction

Chrysomelidae is among the most diverse beetle families, totaling almost 40,000 described extant species in the world [1]. Chrysomelids are known as leaf beetles because most species in this group feed on the green part of the living plant. Some other leaf beetles feed on pollen, flowers, seeds and ant nests debris [2]. The great species diversity of leaf beetles has been ascribed to their co-radiation with the angiosperms [3–5]. Currently, most beetle systematists have reached a general consensus that the family Chrysomelidae includes 12 subfamilies, namely Bruchinae, Cassidinae, Chrysomelinae, Criocerinae, Cryptocephalinae, Donaciinae, Eumolpinae, Galerucinae, Lamprosomatinae, Sagrinae, Spilopyrinae and Synetinae [6,7].

Within the superfamily Chrysomeloidea, the Cerambycidae are often recovered as the sister group of Chrysomelidae [8–11]. The monophyly of Chrysomelidae was well supported by morphological [9] and molecular [10–14] studies. Although a number of previous phylogenetic studies have contributed significantly to our understanding of the evolution of leaf beetles [2,3,5,6,8–10,13–27], the interrelationships of subfamilies in Chrysomelidae remain incompletely resolved.

**Fig 1:**
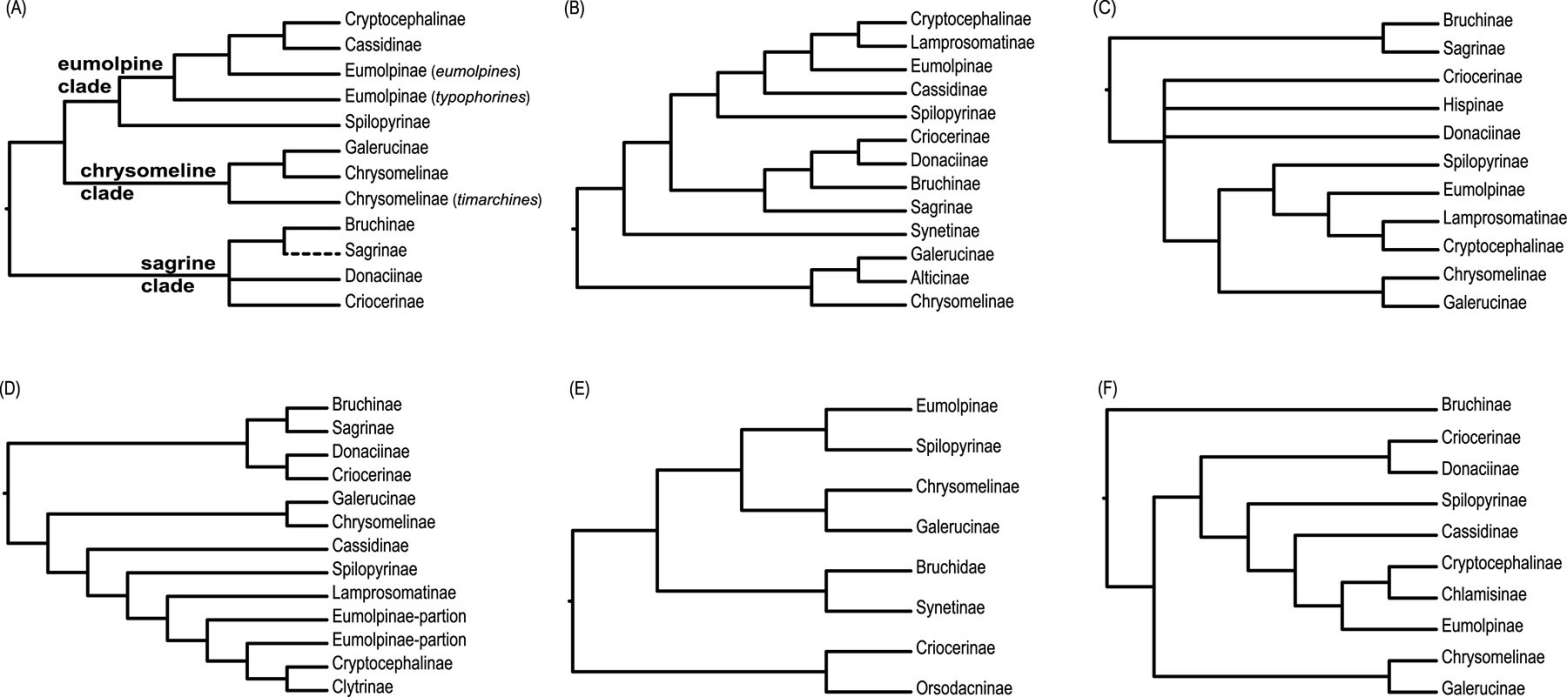
Representations of previous hypotheses for the subfamily-level relationships within Chrysomelidae inferred from morphologically-based or molecular studies by authors cited. (A) Gómez-Zurita at al. (2008) [13] based on molecular data; (B) Nie at al. (2020) [22] based on molecular data; (C) Reid (2000) [26] based on morphological data; (D) Bocak at al. (2014) [11] based on molecular data; (E) Jolivet at al. (2008) [22] based on morphological data; (F) Song at al. (2018) [27] based on molecular data.

Gómez-Zurita et al. [12,13] conducted the comprehensive phylogenetic studies of the basal relationships in the Chrysomelidae, based on two nuclear (*18S* and *28S* rDNA) and mitochondrial (*rrnL*) gene fragments for 167 taxa covering most major lineages and relevant outgroups. The Chrysomelidae was subdivided into three major subfamily groups: “sagrine” (Criocerinae, Donaciinae, Sagrinae and Bruchinae), “eumolpine” (Spilopyrinae, Eumolpinae, Cryptocephalinae and Cassidinae) and “chrysomeline” (Chrysomelinae and Galerucinae), with the basal “sagrine” as sister to the “eumolpine” plus “chrysomeline” clades [13] (Fig 1A). Within the “eumolpine” clade, Cassidinae was sister to Cryptocephalinae [13]. Although the three main chrysomelid lineages were distinguished based on the findings of the study by Gómez-Zurita et al. [13], the key nodes received no significantly statistical support across inference methods.

In the most recent molecular study of Nie et al. [14] (Fig 1B), most of the basal relationships are established, but some remain unclear. The relationships among the three chrysomelid main clades (i.e., sagrines, eumolpines, chyrsomelines) differed across the tree inference methods [14]. the reconstructions of basal relationships were complicated by the non-monophyly of sagrines and eumolpines (e.g., the RAxML tree in Fig 1 of Nie et al. [14]). In addition, the placements of Synetinae and Sagrinae were unresolved (PP = 0.51 and 0.84 in Fig 2 of Nie et al. [14], respectively). Many fundamental questions about Chrysomelidae systematics need to be addressed by additional sampling of taxa and characters.

The subfamily Bruchinae, with about 1700 known species [28,29], are specialized internal feeders of bean seeds. This group of beetles has traditionally been treated as a separate family Bruchidae [29–34]. Numerous phylogenetic studies converged on supporting the group as a chrysomelid subfamily [8–11,14,35]. Within Chrysomelidae, the phylogenetic placement of Bruchinae varied between analyses. Reid [25,26] recovered Sagrinae as the sister group of Bruchinae based on morphological characters (Fig 1C). Farrell and Sequira [15], based on a combined analysis of molecular (*18S* rDNA) and morphological data, also retrieved a sister group relationship between Bruchinae and Sagrinae. In a multi-gene analysis of Hunt et al. [8], Bruchinae was sister to the clade Donaciinae + Criocerinae. In the ML tree of Gómez-Zurita et al. [12], Synetinae was placed as the sister group of Bruchinae. In the further analyses based on multi-locus ribosomal RNA data [13] (Fig 1A), the relative position of Bruchinae remained unclear, though it was placed in a basal “sagrine” clade also containing Donaciinae, Criocerinae and Sagrinae. The morphological study of Lawrence et al. [9] grouped Bruchinae and Cryptocephalinae in a clade. Bocak et al. [11] also recovered a sister-group relationship of Bruchinae to Sagrinae (Fig 1D). In the molecular study of McKenna et al. [10], Bruchinae and Criocerinae clustered together. Some recent analyses, based on the mitogenome sequence data [14,27], recovered Bruchinae as sister to all remaining Chrysomelidae. As reviewed above, most of previous studies have tended to recover a close relationship between Bruchinae and Sagrinae [11,13,15,25,26].

An earlier study suggested a close relationship of the Cassidinae with the “sagrine” clade [3]. In the study of Duckett et al. [36], Cassidinae was recovered as the sister group of the clade Chrysomelinae + Galerucinae. In the study of Hunt et al. [8], the sister group Cassidinae + Hispinae was placed in an intermediate position between the clade Bruchinae + (Criocerinae + Donaciinae) and the clade Sagrinae + ((Galerucinae + Chrysomelinae) + ((Lamprosomatinae + (Cryptocephalinae + Eumolpinae))). Marvaldi et al. [37], based on the secondary structural information of *18S* and *28S* rDNA, also recovered Cassidinae as the sister group of Cryptocephalinae. In the morphological analysis of Lawrence et al. [9], Cassidinae was placed as sister to all other Chrysomelidae. Bocak et al. [11] recovered Cassidinae in a more derived position and as the sister group of a clade including Spilopyrinae, Lamprosomatinae, Eumolpinae, Cryptocephalinae and Clytrinae.

Besides the phylogenetic placements of Bruchinae and Cassidinae, the affinity of Alticinae relative to Galerucinae is another focus of debate on the higher-level phylogeny of Chrysomelidae. Traditionally, based on the presence of the jumping apparatus, flea beetles were distinguished from the closely related Galerucinae [38–40] and considered as an independent subfamily (Alticinae). Several recent studies suggested Alticini as a tribe within Galerucinae s.l. [36,41]. The studies of Ge et al. [16,17] based on the combined analyses of multi-locus sequence data (*18S*, *28S* rDNA, *rrnL* and *cox1*) and morphological characters showed that the traditionally defined Alticinae or Alticini was non-monophyletic. The metafemoral spring is prone to convergence and not sufficient for classification at the subfamily level [17]. In more recent studies by Nie et al. [14,24], the placements of some genera have been transferred between Alticinae and Galerucinae. As a result, two reciprocally monophyletic lineages corresponding to the subfamily level were recognized. Thus, in the new classification of the chrysomelid subfamilies, flea beetles can be classified as a separate subfamily. In this study, we follow the new definition of Alticinae as Nie et al. [14,24].

Mitogenome as a class of molecular marker has been demonstrated to be informative in resolving higher-level phylogeny of Chrysomelidae [14,24,42]. Recently, next-generation sequencing has been effectively used for phylogenetic studies in Coleoptera [14,27,43,44]. This has resulted in rapid increase in the number of mitogenomes of leaf beetles. As of June 2020, there are more than 300 complete or partial mitogenome sequences of Chrysomelidae published in GenBank. In this study, we obtained 16 new mitogenomes of Chrysomelidae by using the next-generation sequencing method.

Given the above-outlined uncertainties in the phylogenetic relationships of the subfamilies of Chrysomelidae, we attempt to resolve the major lineages of the group using the expanded mitogenome sequence data. Specifically, we aim to (1) assess the validity of the subfamily groups of [13], and (2) investigate the placements of Bruchinae, Spilopyrinae, Cassidinae, and *Timarcha*.

## Materials and methods

### Ethics statement

No specific permits were required for the insect specimens collected for this study. These specimens were collected on the roadside of Jigong Mountain tourist attraction. The field studies did not involve endangered or protected species. The sixteen insect species sequenced are all common beetle species in China and are not included in the ‘‘List of Protected Animals in China”.

### Taxon sampling and DNA extraction

The focus of this paper was to recover the relationships between subfamilies in Chrysomelidae, therefore, 205 species representing all 13 recognized subfamilies were included. This represents the most comprehensive taxon sampling of mitogenomes for Chrysomelidae to date (S1 Table). For outgroup taxa, we included five species from the family Cerambycidae.

A total of 16 miotgenomes were newly sequenced in this study. The DNA-grade tissue samples were collected by authors in Jigong mountain (N31°48′42.53″, E114°05′43.10″), Henan province, China in July 2016. Specimen identification were conducted by checking adult morphological characters, and molecular identification through blasting mitochondrial *cox1* gene fragments in online identification tool of BOLD systems (Barcode Of Life Database: http://www.boldsystems.org–‘Identification’ section), and by the Standard Nucleotide BLAST in NCBI. Voucher specimens for all newly sequenced taxa are deposited at the Entomological Museum of Henan Agricultural University. The insects were preserved in 100% ethanol and stored at -20°C before DNA extraction. Whole genomic DNA was extracted from legs or thoracic muscle of single specimens with the TIANamp Genomic DNA Kit (TIANGEN BIOTECH CO., LTD), following the manufacturer’s instructions.

### Library preparation, illumina sequencing and genome assembly

Five libraries including the single species genomic DNA were constructed, namely the individual species library. Approximately 1 Gbp raw data were generated for the individual species library. In addition, seven libraries included multiple species, namely the multiplex sample library. Besides the sequenced leaf beetle species, other 20 distantly related species with equimolar amounts of DNA were pooled into a library, respectively. Approximately 20 Gbp raw data were generated for each of the library including multiple species. For both kinds of libraries, genomic DNA was sonicated to 300 bp using Covaris S220 focused-ultrasonicator (Covaris Inc.), according to Illumina’s protocol. Genomic libraries were constructed using an Illumina TruSeq TM DNA Sample Prep Kit (Illumina, San Diego, CA, USA). Genome sequencing was performed on an Illumina HiSeq 2500 platform (Beijing Novogene Bioinformatics Technology Co., Ltd, China), using 150 bp paired-end run.

The raw reads were demultiplexed and concatenated. The low-quality reads, low-quality ends, and adapter sequences were trimmed using NGS QC Toolkit [45]. The clean reads were used in the genome assembly. We used IDBA-tran [46] to conduct the *de novo* assembly. The parameters are set to the minimum size of contig of 200, an initial k-mer size of 41, an iteration size of 10, and a maximum k-mer size of 91.

### Mitogenome assembly and annotation

The contigs generated by IDBA-tran were used to construct a Blast database. We used the pre-sequenced mitochondrial gene fragments (*cox1*, *cob* and *rrnS*) to bait the associated mitochondrial contigs by performing the local Blast searches. The primers used to amplify the bait gene sequences are the same as those in the study of Song et al. [47].

The preliminary mitogenome annotations were conducted in MITOS web [48], under default settings and the invertebrate genetic code for mitochondria. The start codon, stop codon and length of each protein-coding genes (PCGs), and the rRNA gene boundaries were refined by alignment against the published chrysomelid beetle mitogenome sequences in GenBank. The tRNA secondary structures were predicted in MITOS web. The new mitogenome sequences generated in this study are deposited to GenBank with accession numbers: MW035611-MW035626.

### Sequence alignment

PCGs were individually aligned using MAFFT [49] in the TranslatorX [50] server. Ambiguously aligned sites were removed using Gblocks v 0.91 [51], with the options for a less stringent selection. Each of tRNA and rRNA genes was aligned in MAFFT server, with the “E-INS-i” strategy. The poorly aligned regions were trimmed using Gblocks v 0.91. The alignments of different gene types were concatenated together with FASconCAT_v1.0 [52]. Three different concatenated matrices were compiled: (1) PCG_nt (nucleotide sequences of 13 PCGs), (2) PCG_aa (amino acid sequences of 13 PCGs), and (3) PCGnt+RNA (combined nucleotide sequences of 13 PCGs, 22 tRNA genes and two rRNA genes). In order to reduce the impact of saturation and compositional heterogeneity, we recoded the amino acid matrix using the Dayhoff 6-states alphabet corresponding to amino acid groups [53,54] to construct the dataset PCGaa_Dayhoff.

Sequence substitution saturation tests on different data partitions were performed in DAMBE 5 [55] using Xia’s method [56]. The heterogeneity of sequence divergence within various datasets was analyzed using AliGROOVE [57], with the default sliding window size. Alignments used in the phylogenetic analyses of this article are provided in the S1 File.

### Phylogenetic analyses

Two different inference approaches were employed to conduct tree searches: Maximum Likelihood (ML) with IQ-TREE [58,59], and Bayesian inference (BI) employing the site-heterogeneous CAT series models [60] with PhyloBayes MPI [61]. All phylogenetic analyses were performed on the CIPRES Science Gateway [62].

For the ML analyses, we used ModelFinder [63] to select the best-fitting partition schemes and the corresponding substitution models (S2 Table). The corrected Akaike information criterion (AICc) was applied to each of the datasets. For the nucleotide alignments of PCGs, the data blocks were defined by gene and by codon. All the 22 tRNA genes were set to be a single partition, while each of the two rRNA genes were defined as the separate partitions. For the amino acid alignment PCG_aa, the data blocks were defined by gene. Node supports were evaluated using 10,000 ultrafast bootstrap replicates. The bootstrap supports (BS) of ≥ 70 were considered to be strong support values for tree nodes, following Hillis & Bull [64].

For the BI analyses, we used the CAT-GTR model for the nucleotide alignments (PCG_nt and PCG_nt+RNA) and the CAT model for the amino acid alignments (PCG_aa and PCGaa_Dayhoff). Two independent Markov chain Monte Carlo (MCMC) chains were run for each alignment, and constant sites were removed. Minimum number of cycles was set to 20,000. The “maxdiff” value being less than 0.3 was considered to be acceptable. That is, the two chains had satisfactorily converged. The initial 25% trees of run were discarded as burn-in, and the majority-rule consensus tree was calculated from the saved trees. The tree nodes having the posterior probabilities (PP) of ≥ 0.95 were considered strongly supported [65,66].

The four-cluster likelihood-mapping (FcLM) approach [67] was employed to study the amount of phylogenetic information contained in the amino acid dataset PCG_aa. Simultaneously, we explored the hypotheses of the deeper phylogenetic relationships in Chrysomelidae through FcLM analysis. The FcLM analysis was conducted using IQ-TREE with the models as in ML tree searches.

## Results

### Genome sequencing and characteristics of mitogenomes

**Fig 2:**
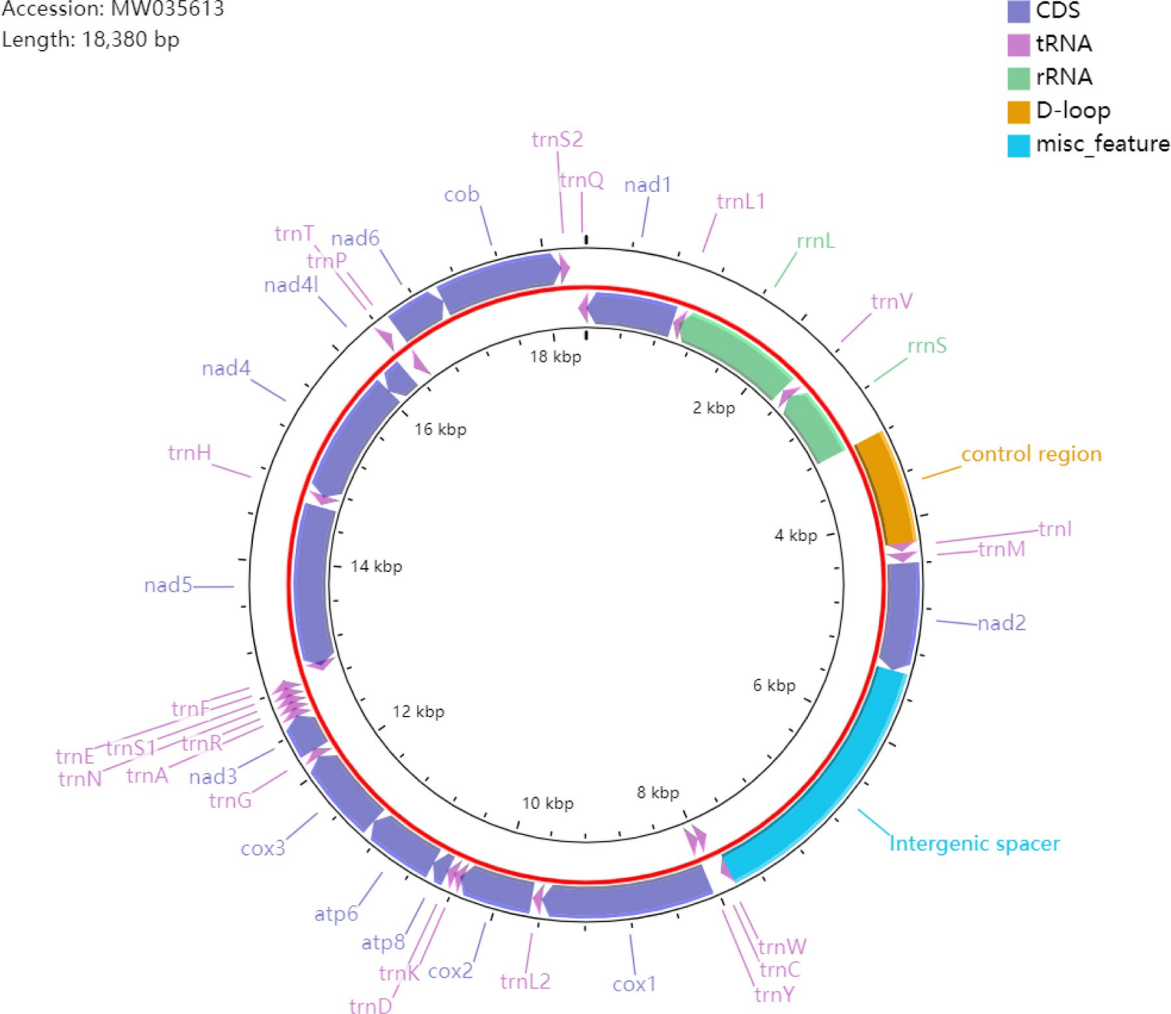
Organization of the mitogenome of *Callosobruchus maculatus*.

Eight new mitogenomes with a genome size of more than 15 kb included the full set of 37 mitochondrial genes and the complete control region (e.g., the *Callosobruchus maculatus* in Fig 2). The remaining eight leaf beetles newly sequenced had the partial mitogeomes (often < 10 kb), in which some gene regions and/or the control region were missing due to failure of genome sequencing and assembling. With regard to the completeness of mitogenomes, the individual species library construction presented the better results than the multiple species library construction. In addition, sequencing depth was correlated with the full assembly of mitogenomes. The detailed statistics of the sequencing of mitogenomes using next-generation sequencing technology was shown in Table 1.

**Table 1:** Statistics associated to the sequencing of mitogenomes.

Species Name	Mitogenome Length (bp)	Library Type	Integrity	Total Reads	Mapped Bases	Mean Coverage
*Geinula* sp.	17,696	single species	complete	13,700,313	11,266,237	637
*Labidostomis lucida*	15,948	single species	complete	7,671,316	11,550,900	706
*Leptomona* sp.	13,497	single species	partial	8,900,740	3,149,850	218
*Sangariola fortunei*	16,176	single species	complete	10,914,741	14,950,800	753
*Callosobruchus maculatus*	18,380	single species	complete	9,006,119	7,663,200	366
*Physosmaragdina nigrifrons*	15,618	multiple species	complete	43,840,589	5,835,450	374
*Oulema* sp.	9,811	multiple species	partial	44561478	805950	82
*Cryptocephalus* sp.	15,955	multiple species	complete	44,561,478	4,127,850	259
*Trirhabda* sp.	9,510	multiple species	partial	44,561,478	513,000	54
*Phratora* sp.	9,239	multiple species	partial	29,492,015	903,000	98
*Lema cyanella*	10,038	multiple species	partial	41,673,375	1350,300	100
*Cassida* sp.	9,873	multiple species	partial	41,673,375	670,800	66
*Plagiodera versicolora*	9,073	multiple species	partial	33,990,316	1,356,900	150
*Clitenella fulminans*	9,743	multiple species	partial	33,225,958	633,300	63
*Clytra* sp.	15,763	multiple species	complete	33,225,958	3,018,750	192
*Smaragdina* sp.	16,156	multiple species	complete	32,149,777	2,974,050	182

The majority of the new mitogenomes showed the similar genome organization and gene content with the putatively ancestral insect mitogenome [68,69], with the exception of *C*. *maculatus* and *Plagiodera versicolora*. In *C*. *maculatus*, a large intergenic spacer region (2,070 bp) was identified between *nad2* and *trnW* (Fig 2). This also resulted in a lager genome size of *C*. *maculatus* (18,380 bp). In addition, the *trnQ* gene (typically between *trnI* and *trnM*) was translocated to the downstream of *trnS2* on the heavy strand. For the partial mitogenome of *P*. *versicolora*, we detected a tRNA translocation for the *trnL1* (typically between *nad1* and *rrnL*), which was translocated to the position between *trnY* and *cox1*.

### Phylogenetic inference

**Fig 3:**
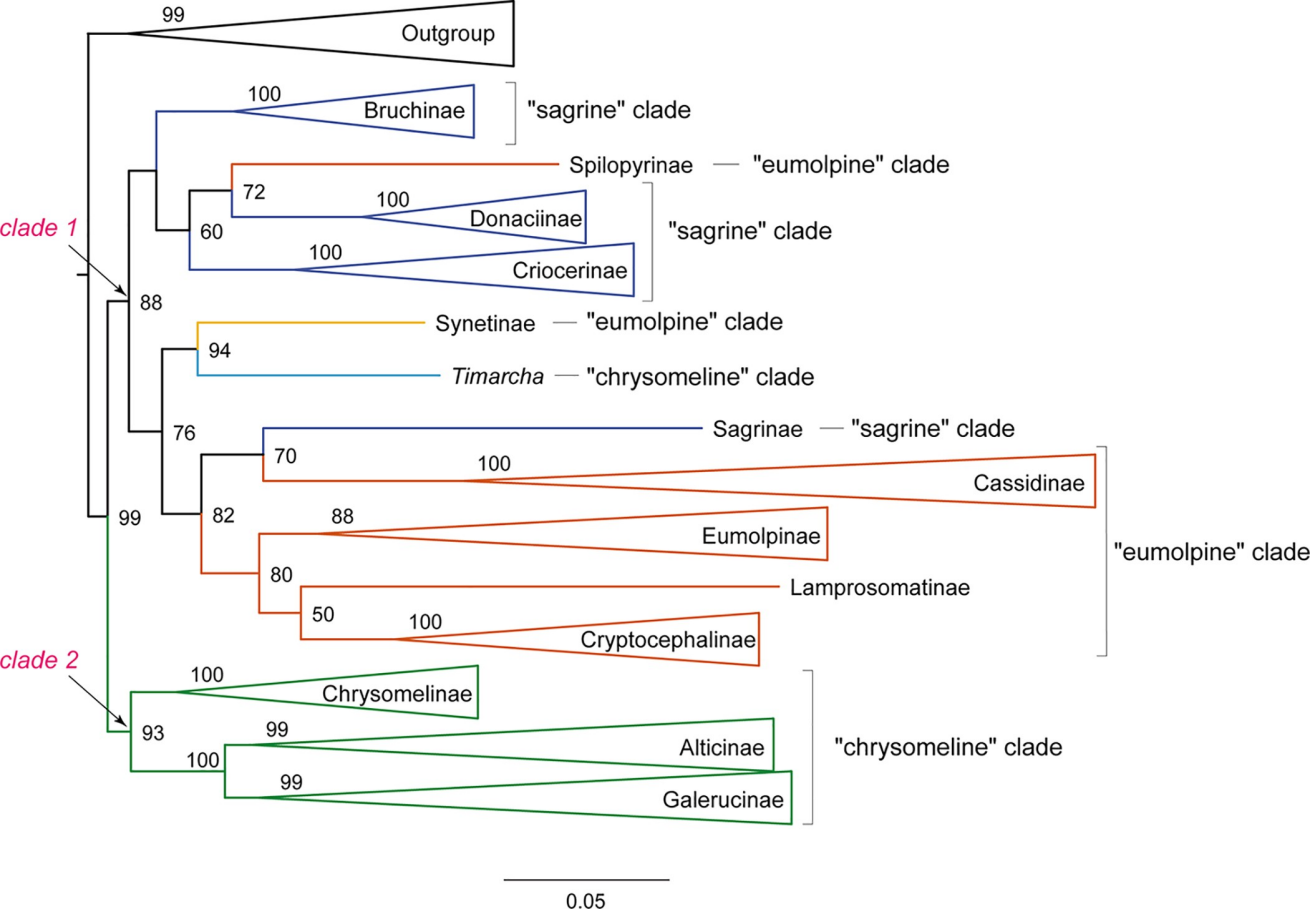
ML tree inferred from the dataset PCGaa_Dayhoff using IQ-TREE under the MK+FQ+I+G4 model. Lineages have been collapsed for clarity. The lengths of the triangles correspond to the longest terminal branches in the collapsed lineages. Node numbers show the bootstrap support values. The full tree with all branches is available in S1 Fig.

**Fig 4:**
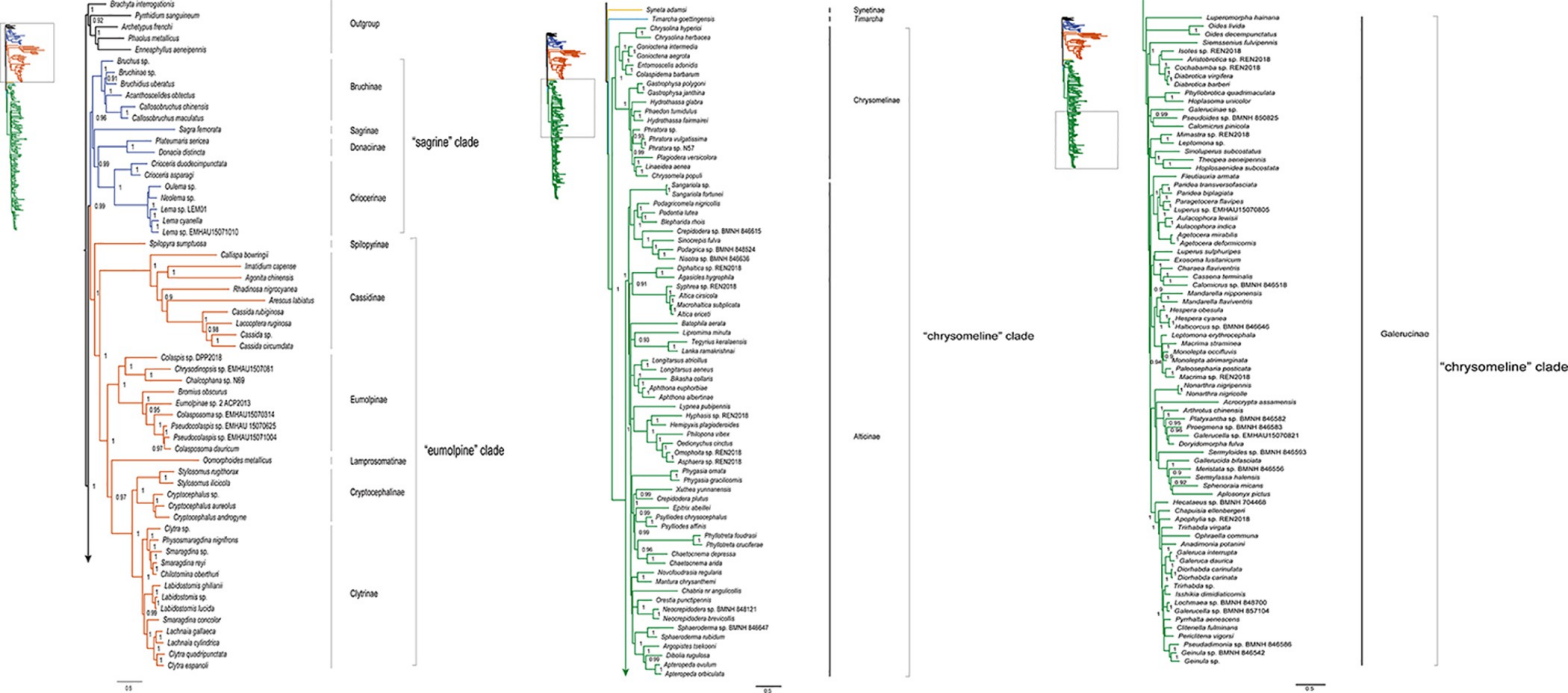
Bayesian tree inferred from the dataset PCG_aa using PhyloBayes under the site-heterogeneous CAT model. Node numbers show the posterior probability values.

**Fig 5:**
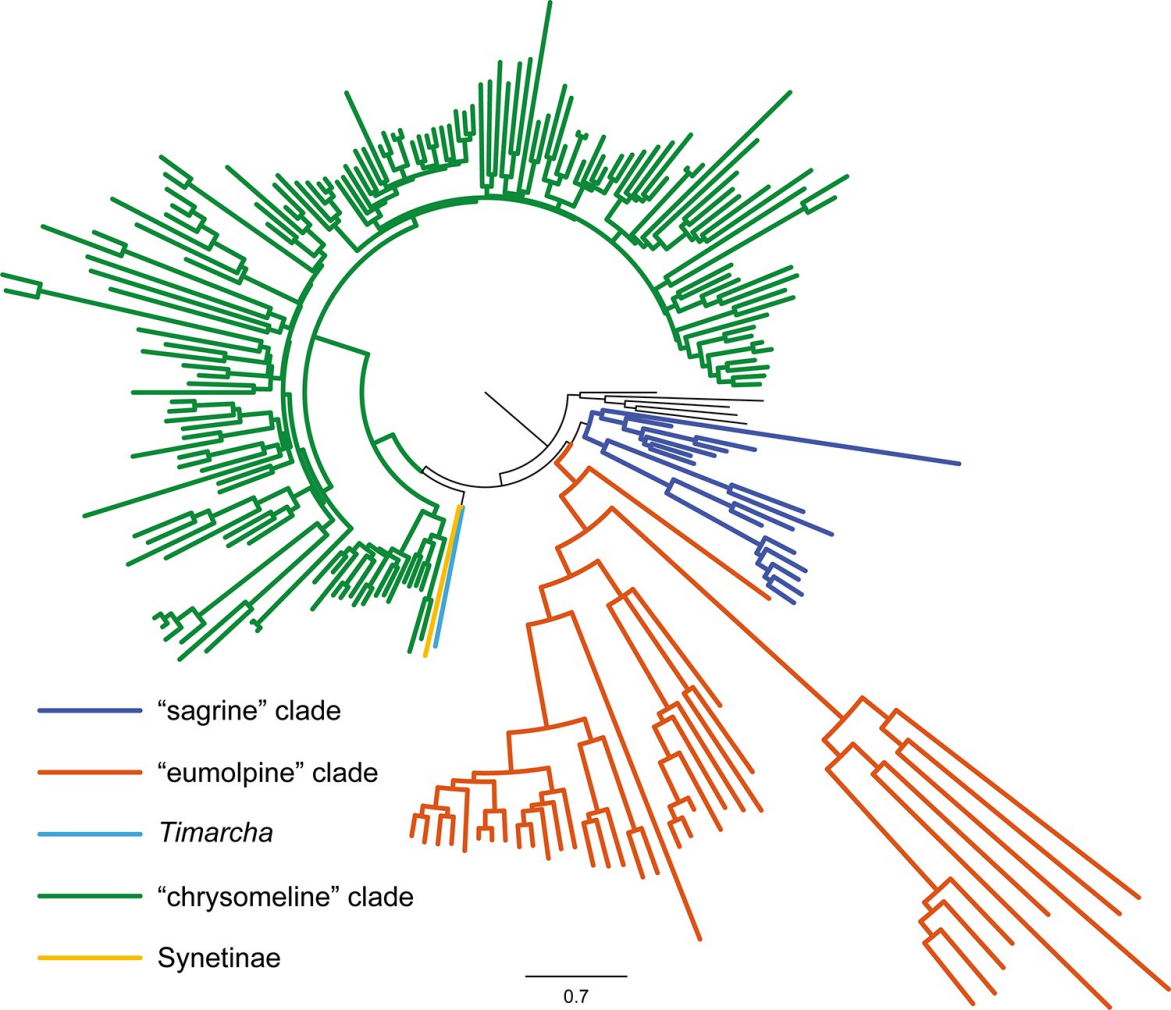
Bayesian tree inferred from the dataset PCGnt+RNA using PhyloBayes under the site-heterogeneous CAT-GTR model. The full tree with all branches is available in S8 Fig.

To compare with the prior studies, we used the delimitation of three major sublineages (i.e., the “chrysomelines”, “eumolpines” and “sagrines”) of Gómez-Zurita et al. [13] (Fig 1A) to discuss the subfamily relationships within Chrysomelidae. Our analyses constantly recovered a monophyletic Chrysomelidae, with strong nodal support (BP ≥ 99, PP ≥ 0.96) (Figs 3–5 and S1–S8 Figs). Chrysomelidae was subdivided into two main clades: (clade 1) Bruchinae, Sagrinae, Donaciinae, Synetinae, Criocerinae, Spilopyrinae, Cassidinae, Eumolpinae, Lamprosomatinae and Cryptocephalinae; and (clade 2) Chrysomelinae, Alticinae and Galerucinae. In some analyses, Synetinae was placed in the clade 1. Other discrepancies among analyses were restricted to the relationships of subfamilies in the “sagrine” and “eumolpine” clades, in which the branching order varied depending on the datasets and methods.

For the ML analyses, we recovered a tree topology comparable with the hypothesis proposed by Gómez-Zurita et al. [13], when recording the amino acid matrix with Dayhoff categories (Figs 3 and S1). The ML tree with Dayhoff recoding had the log-likelihood score of -218718.087, which was higher than other ML trees. In clade 1, we recovered a deep subdivision of analysed taxa into two groups corresponding to the “sagrine” and “eumolpine” clades. Differences between the Dayhoff ML tree and the hypothesis of Gómez-Zurita et al. [13] lied mainly in the placements of Sagrinae and Spilopyrinae. In the Dayhoff ML tree, Spilopyrinae was nested within the “sagrine” clade and as sister to Donaciinae (BP = 72). Sagrinae represented by a single species of *Sagra femorata* appeared as sister to Cassidinae, both of which were sister to the major “eumolpine” clade. Last but not least, the “chrysomeline” clade was sister to the “sagrine” plus “eumolpine” clade. In the remaining ML analyses, Bruchinae was sister to all other taxa of the clade 1. The “eumolpine” clade including Spilopyrinae, Cassidinae, Eumolpinae, Lamprosomatinae and Cryptocephalinae was retrieved as monophyletic. However, the “sagrine” clade was not supported with respect to Bruchinae (e.g., S2 Fig).

The BI analyses under the empirical site-heterogeneous CAT mixture model provided more clearly resolved relationships in Chrysomelidae. In three out of four BI analyses, each of the three subfamily groups proposed by Gómez-Zurita et al. [13] were strongly supported (e.g., PCG_aa BI tree in Fig 4). The phylogenetic placement of Synetinae remained unresolved across the BI analyses. The BI tree from the dataset PCG_aa recovered Synetinae as sister to the “chrysomeline” clade, but without the significantly statistical support (PP = 0.8).

The similar situation occurred in the BI tree from the dataset PCGnt+RNA (Fig 5). For the inter-subfamily relationships in the “eumolpine” clade, the BI trees were concordant with the majority of ML trees. The following subfamily relationships were consistently found: (Spilopyrinae + (Cassidinae + (Eumolpinae + (Lamprosomatinae + Lamprosomatinae)))). Within the “sagrine” clade, the BI tree from the dataset PCG_aa placed Bruchinae as the most basal lineage, followed by Sagrinae, and Donaciinae + Criocerinae. In contrast, the alternative branching pattern was retrieved in the BI tree from the nucleotide datasets PCG_nt and PCGnt+RNA. In both analyses, Sagrinae formed a sister group of Bruchinae, which in turn was sister to the clade Donaciinae + Criocerinae. The “sagrine” clade was recovered as the sister group of the “eumolpine” clade in all BI analyses except for that with Dayhoff recoding.

**Fig 6:**
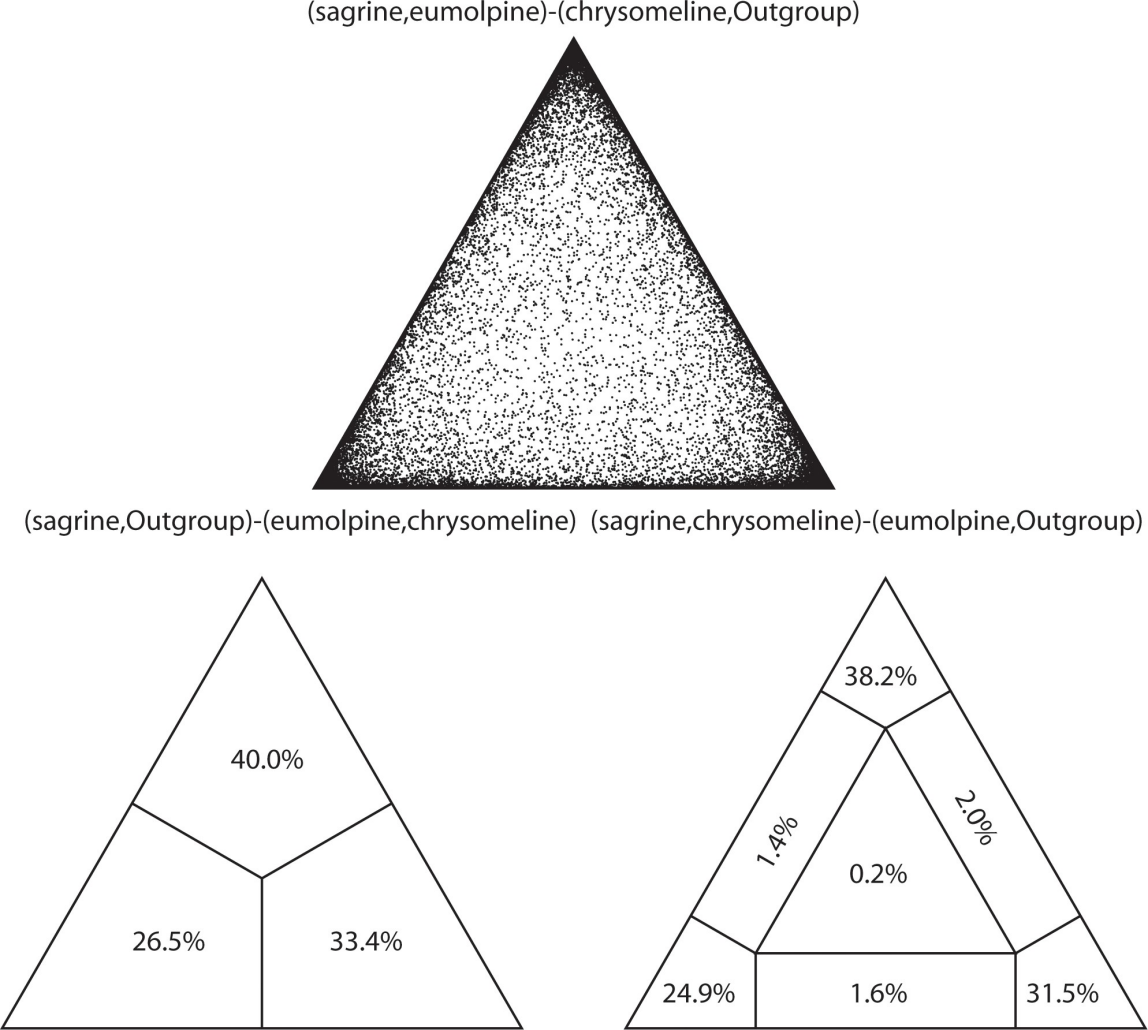
Results obtained from the four-cluster likelihood-mapping analysis based on the dataset PCG_aa showing conflicting signal for the alternative hypotheses. The above triangle picture shows the possible relationships of four clusters. The below triangle picture on the left is the three posterior probabilities for the three possible unrooted trees of four clusters. The below triangle pictures on the right shows the seven areas supporting different evolutionary information from the dataset PCG_aa.

The FcLM analysis testing the relationships among the three major chrysomelid clades showed conflicting phylogenetic signal present in the dataset PCG_aa. Support for the relationship between the “sagrine”, “eumolpine” and “chrysomeline” clades was divided, though the highest percentage of data points fell in favor of a branching pattern of ((“sagrine” + “eumolpine”) + “chrysomeline”) (38.2%, in Fig 6).

## Discussion

Mitochondrial DNA as a phylogenetic marker has its potential shortcomings, for example, the substitutional saturation (at a single site or some gene regions) [70] and lineage-specific compositional heterogeneity [71]. Saturation tests showed that the third codon positions of PCGs, as well as the *rrnL* and *rrnS* gene regions were saturated in our data sets (S3 Table).

**Fig 7:**
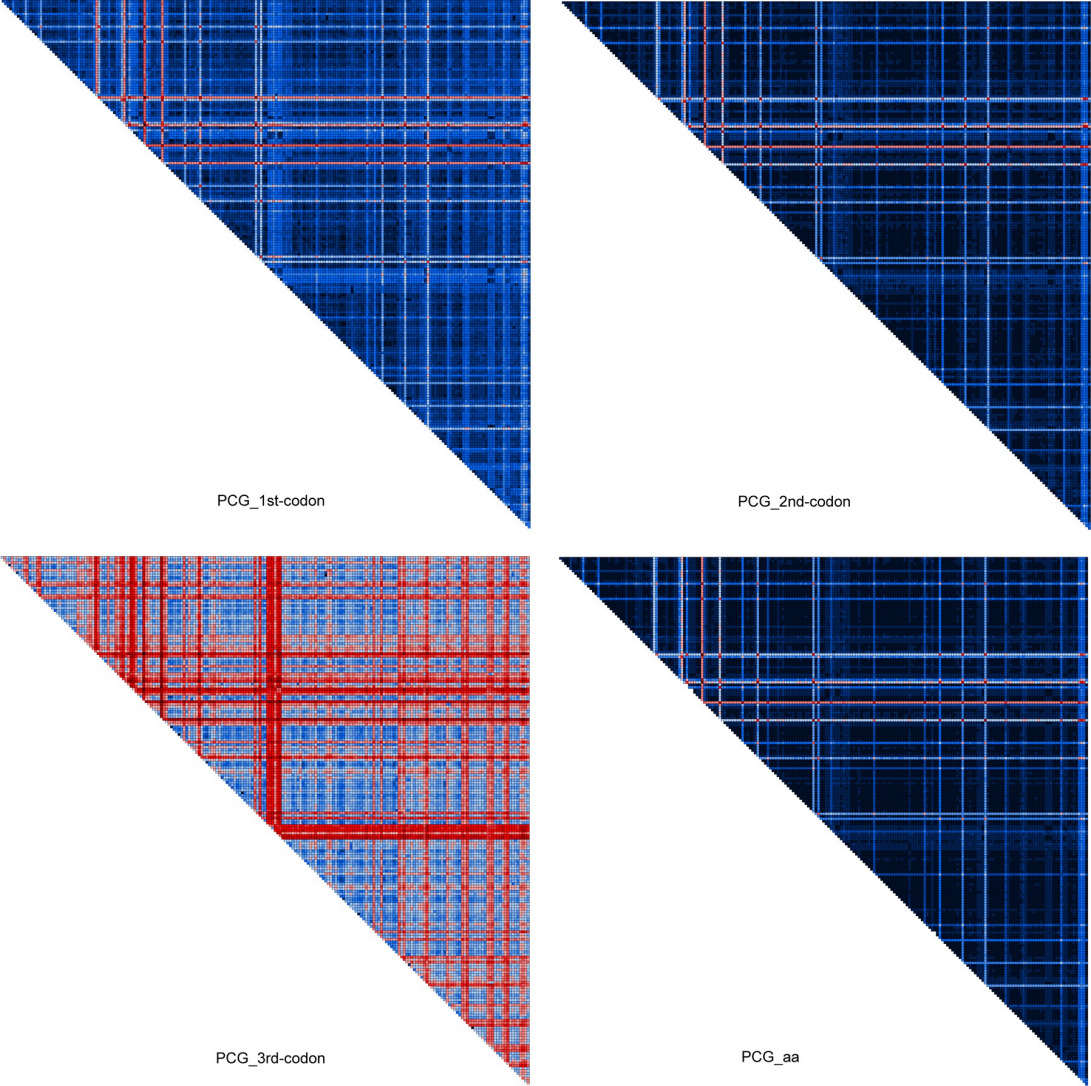
AliGROOVE heat maps of pairwise sequence comparisons for the data partitions PCG_1st-codon, PCG_2nd-codon, PCG_3rd-codon and the dataset PCG_aa. The AliGROOVE graph shows the mean similarity scores between sequences. AliGROOVE scores range from −1 (indicating great difference in rates from the remainder of the data set, i.e. red coloring implies the significant heterogeneity) to +1 (indicating rates match all other comparisons).

The sequence heterogeneity analyses indicated that the greatest degree of heterogeneity occurred at third codon positions of PCGs, and that some heterogeneity also occurred in the other data partitions (Figs 7 and S9).

In this study, most phylogenetic analyses conducted on the concatenated matrices revealed a well-supported topology for deep nodes in the chrysomelid tree of life. The monophyly of Chrysomelidae was strongly supported in all analyses. This was concordant with previous studies [9–14]. Nevertheless, the relationships among subfamilies in Chrysomelidae varied across analyses. The major concerns in phylogenetic reconstruction based on the insect mitogenomes have been the substitutional saturation and compositional heterogeneity as mentioned above. Because both factors may have negative effects on the accuracy of the reconstructed phylogeny. To reduce the effect of substitution saturation, we translated nucleotide sequences into amino acid sequences. Furthermore, we recoded the matrix of amino acids into Dayhoff categories, by which the 20 character states of amino acids were reduced down to six states [53]. As a result, the sequence heterogeneity was reduced greatly by Dayhoff recoding. In the ML analysis with Dayhoff recoding under the homogeneous model, three main clades recovered largely matched the three major subfamily groups of Chrysomelidae proposed by Gómez-Zurita et al. [13]. In contrast, the ML analysis of PCG_aa without Dayhoff recoding and the ML analyses with nucleotide datasets did not produce a clear relationship of the subfamilies corresponding to the “sagrine” and “eumolpine” clades. The discrepancies across datasets in the ML analyses demonstrated that reducing the heterogeneity of mitogenome data can improve the recovery of a reasonable relationship in Chrysomelidae, even under the homogeneous model of evolution.

Under the site-heterogeneous model, nucleotide and amino acid data were basically congruent, with nucleotide datasets also strongly supporting the three major subfamily groups of Chrysomelidae [13]. These results showed that applying the site-heterogeneous model in the BI analyses lessened the effect of compositional heterogeneity. The BI tree based on the amino acid dataset was preferred (Fig 4), due to the strong support for deep nodes. Although each of the “sagrine”, “eumolpine” and “chrysomeline” clades was supported in the majority of BI analyses, their interrelationships conflicted with the study of Gómez-Zurita at al. [13]. In the present study, the “sagrine” clade was always recovered as the sister group of the “eumolpine” clade, together forming the sister group of the “chrysomeline” clade. In contrast, the branching pattern of “sagrine” + (“eumolpine” + “chrysomeline”) was supported in the study of Gómez-Zurita at al. [13]. Result of the FcLM analysis testing the major nodes connecting the three subfamily groups revealed conflicting signal for the inferred relationship on the dataset PCG_aa (Fig 7). Further study is required to provide resolution in nodes of the major subfamily groups, emphasizing denser taxon sampling in the “sagrine” and “eumolpine” clades.

In this study, bruchid seed beetles were consistently recovered as members of Chrysomelidae, consistent with recent phylogenetic analyses focusing on this clade [8–11,14,35]. Thus, the current mitogenome data supported the subfamily rank of Bruchinae within Chrysomelidae. In the “sagrine” clade, Bruchinae was sister to Sagrinae in the BI trees from the datasets PCG_nt (PP = 0.91) and PCGnt+RNA (PP = 0.97). This result was congruent with previous studies [11,15,25,26]. In addition, the sister-group relationship between Donaciinae and Criocerinae [8,11,36] received strongly support in most analyses under both homogeneous and heterogeneous models.

The status of Spilopyrinae was controversial in prior studies [26]. Reid [26] and Marvaldi et al. [37] proposed to elevate Spilopyrini to subfamily rank (as Spilopyrinae). However, Jolivet & Verma [22] placed Spilopyrinae within Eumolpinae (Fig 1E), based on morphological data. Gómez-Zurita et al. [12,13] recovered Spilopyrinae as the most-basal lineage in the “eumolpine” clade. In the present study, seven out of eight phylogenetic analyses retrieved Spilopyrinae as sister to all other “eumolpine” clade. This supported the subfamily status of Spilopyrinae.

The members of *Syneta* were traditionally classified within Eumolpinae as a tribe (Synetini) [15,25]. *Syneta* was placed as an early separated lineage in Eumolpinae [19,26,70]. Some authors have proposed the subfamily rank (Synetinae) for this group and supported the exclusion of *Syneta* from Eumolpinae [22,70]. The close relationship of Synetinae to Eumolpinae was not retrieved in the present study. In the preferred tree (PCG_aa BI tree in Fig 4), Synetinae represented by a single species of *Syneta adamsi* was sister to the “chrysomeline” clade. Most of other analyses retrieved a close affinity of Synetinae to *Timarcha*. Together, these taxa were sister to either the “eumolpine” clade or the “chrysomeline” clade. In fact, the phylogenetic placement of Synetinae was unstable in the previous study [71], because different inference methods produced conflicting results on *Syneta*. Morphological analyses could not address this problem [19,70], either. Based on the current mitogenome data, the phylogenetic position of Synetinae is still ambiguous.

The phylogenetic placement of Cassidinae in Chrysomelidae has been the subject of debate. Wilf et al. [72] hypothesized a single origin of monocot feeding in Chrysomelidae. The monocot feeding groups included the Donaciinae, Cassidinae and Cryptocephalinae. However, the morphological characters uniting the monocot feeding groups were considered to be convergent [13]. In the study of Gómez-Zurita et al. [13], the Cassidinae was kept separate from the other major monocot feeding groups in the “sagrine” clade and as sister to Cryptocephalinae s.l. in the “eumolpine” clade. Our results consistently recovered Cassidinae nested within the “eumolpine” clade and as sister to the clade comprising Eumolpinae, Lamprosomatinae and Cryptocephalinae.

Crownson [1] established the subfamily Clytrinae, which was composed by the tribes Lamprosomatini, Cryptocephalini, Clytrini and Chlamisini. The subsequent studies elevated the former Lamprosomatini to subfamily status (as Lamprosomatinae) [25,73]. The remaining tribes Cryptocephalini, Clytrini and Chlamisini constituted the subfamily Cryptocephalinae [25,73]. The monophyletic Cryptocephalinae was further supported by Gómez-Zurita et al. [13]. In the present study, the sister-group relationship between Cryptocephalinae and Lamprosomatinae was consistently recovered by the mitogenome data. This arrangement supported the hypothesis of Gómez-Zurita et al. [13].

The Chrysomelinae was non-monophyletic with respect to *Timarcha*. This result was consistent with the previous studies [2,13,14,18]. Thus, our analyses reinforced the point that *Timarcha* should be regarded as a separate clade in Chrysomelidae [2,18]. As for Galerucinae and Alticinae, the relationships recovered by the current mitogenome data were consistent with the prior mitogenomic study [14].

## Conclusions

Expanded mitogenome data resulted in the improved resolution of the higher-level phylogeny of Chrysomelidae, as the deep notes having the generally high nodal support values. When we used the mixture heterogeneous CAT model in the BI analyses, the three major subfamily groups recognized by Gómez-Zurita et al. [13] were strongly supported (Fig 3, sagrine clade: PP = 0.96, eumolpine clade: PP = 1, chrysomeline clade: PP = 1). In addition, the phylogenetic placements of Bruchinae, Spilopyrinae and Cassidinae were resolved with confidence. Despite this, we acknowledged the potential pitfalls of mitochondrial DNA sequences in reconstructing the phylogenetic relationships in Chrysomelidae. The heterogeneous sequence divergence may lead to conflicting signals for the alternative hypothesis of the interrelationships among three major subfamily clades of Chrysomelidae. The placement of *Syneta* remained questionable. Further mitogenome studies should sequence more species from *Syneta* and other taxa from the “sagrine” and “eumolpine” clades.

## Supporting information

S1 Fig ML tree inferred from the dataset PCGaa_Dayhoff using IQ-TREE. (PDF)

S2 Fig ML tree inferred from the dataset PCG_aa using IQ-TREE. (PDF)

S3 Fig ML tree inferred from the dataset PCG_nt using IQ-TREE. (PDF)

S4 Fig ML tree inferred from the dataset PCGnt+RNA using IQ-TREE. (PDF)

S5 Fig Bayesian tree inferred from the dataset PCGaa_Dayhoff using PhyloBayes. (PDF)

S6 Fig Bayesian tree inferred from the dataset PCG_aa using PhyloBayes. (PDF)

S7 Fig Bayesian tree inferred from the dataset PCG_nt using PhyloBayes. (PDF)

S8 Fig Bayesian tree inferred from the dataset PCGnt+RNA using PhyloBayes. (PDF)

S9 Fig AliGROOVE heat maps of pairwise sequence comparisons for the data partitions of *rrnL*, *rrnS* and tRNA. (PDF)

S1 Table Taxa included in this study. (XLSX)

S2 Table The best-fitting partition schemes and the corresponding substitution models selected by ModelFinder. (XLSX)

S3 Table Saturation tests conducted in DAMBE. (XLSX)

S1 File Alignments used in the phylogenetic analyses of this article. (ZIP)
