# Supplementary figures and images for: Higher-level phylogeny of Chrysomelidae based on expanded sampling of mitogenomes
Source: PLoS One. 2022 Jan 21;17(1):e0258587. doi: 10.1371/journal.pone.0258587 (PMC8782406; doi:10.1371/journal.pone.0258587)

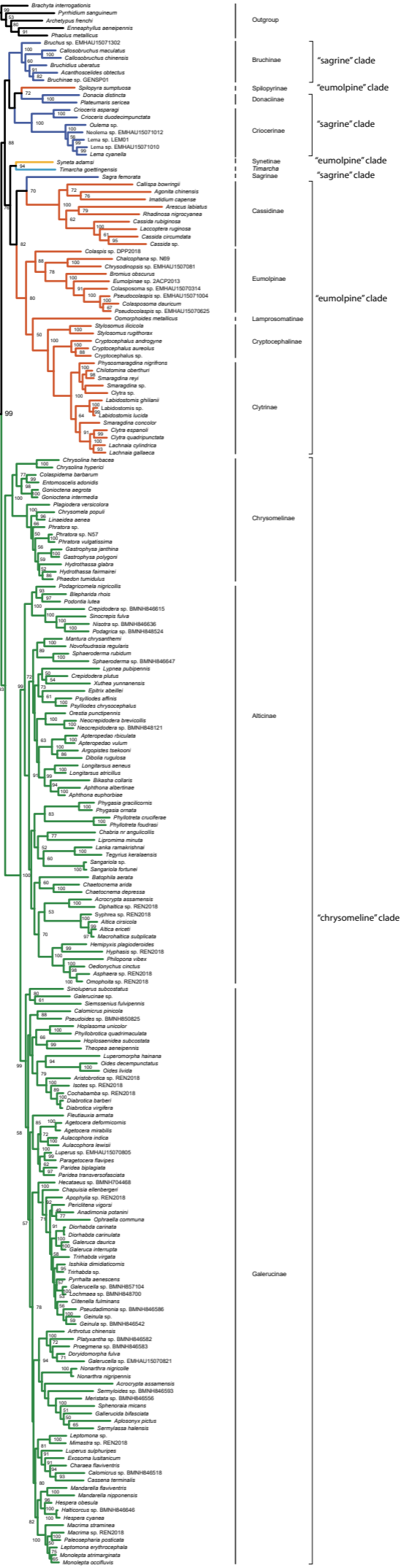

Supplement: S1 Fig — (PDF) [file pone.0258587.s001.pdf]

PCG\_aa IQ-TREE

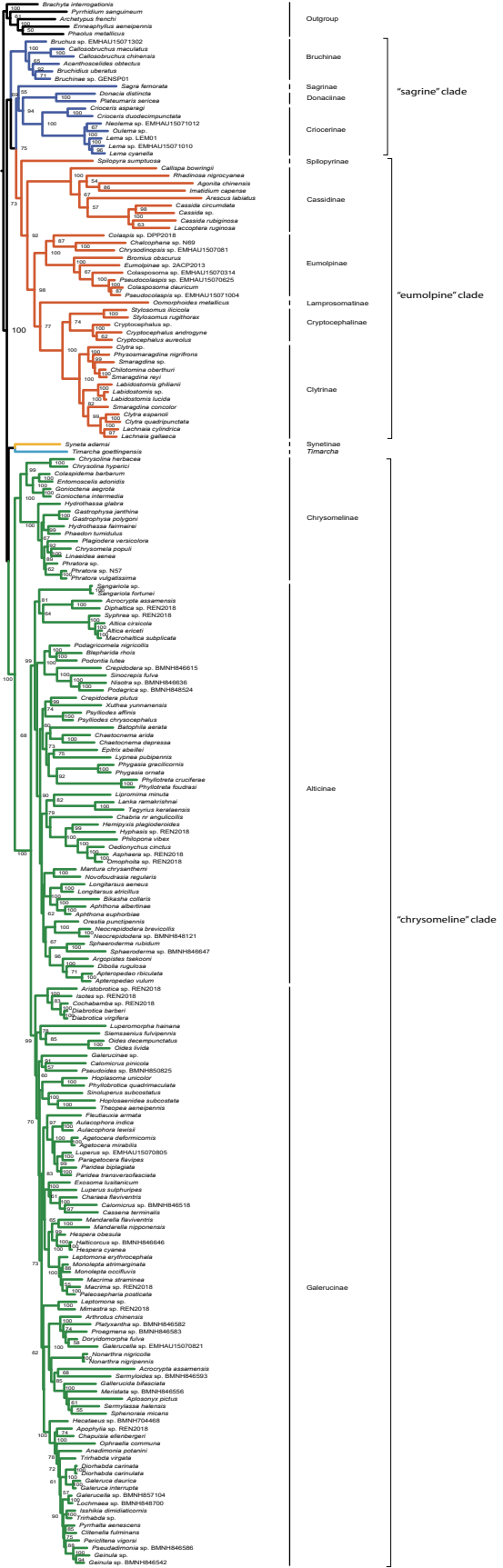

Supplement: S2 Fig — (PDF) [file pone.0258587.s002.pdf]

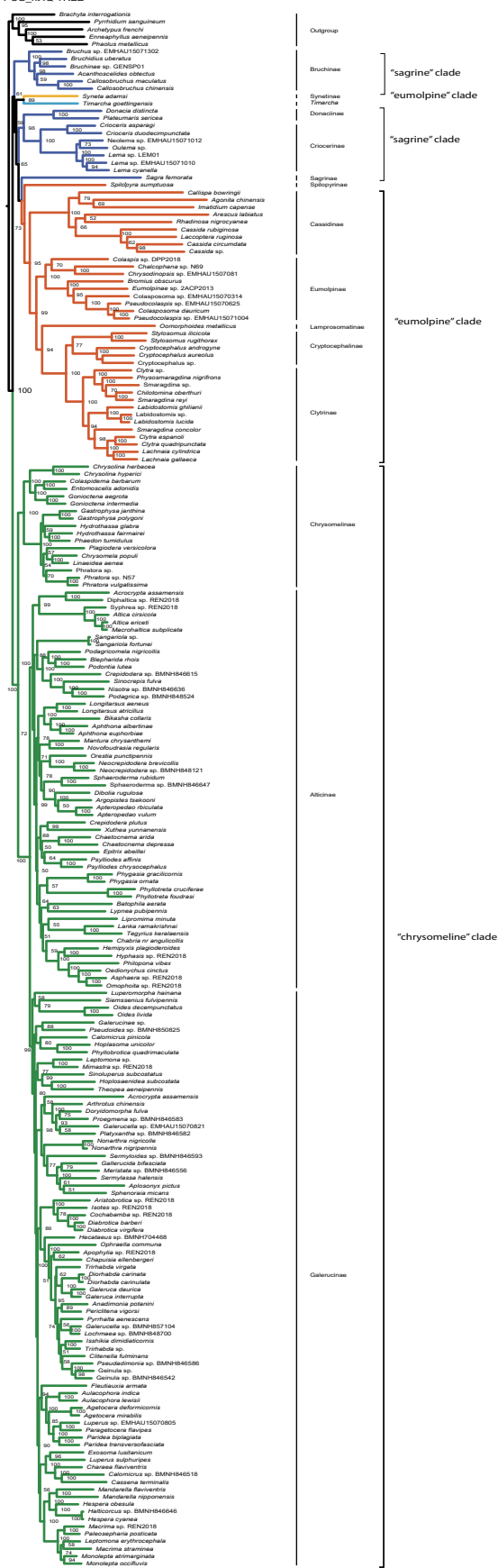

Supplement: S3 Fig — (PDF) [file pone.0258587.s003.pdf]

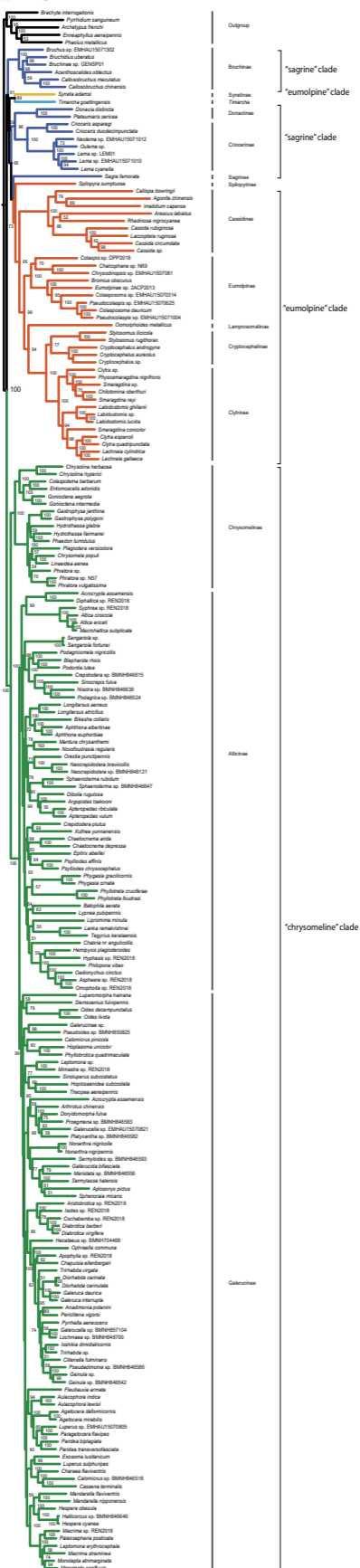

Supplement: S4 Fig — (PDF) [file pone.0258587.s004.pdf]

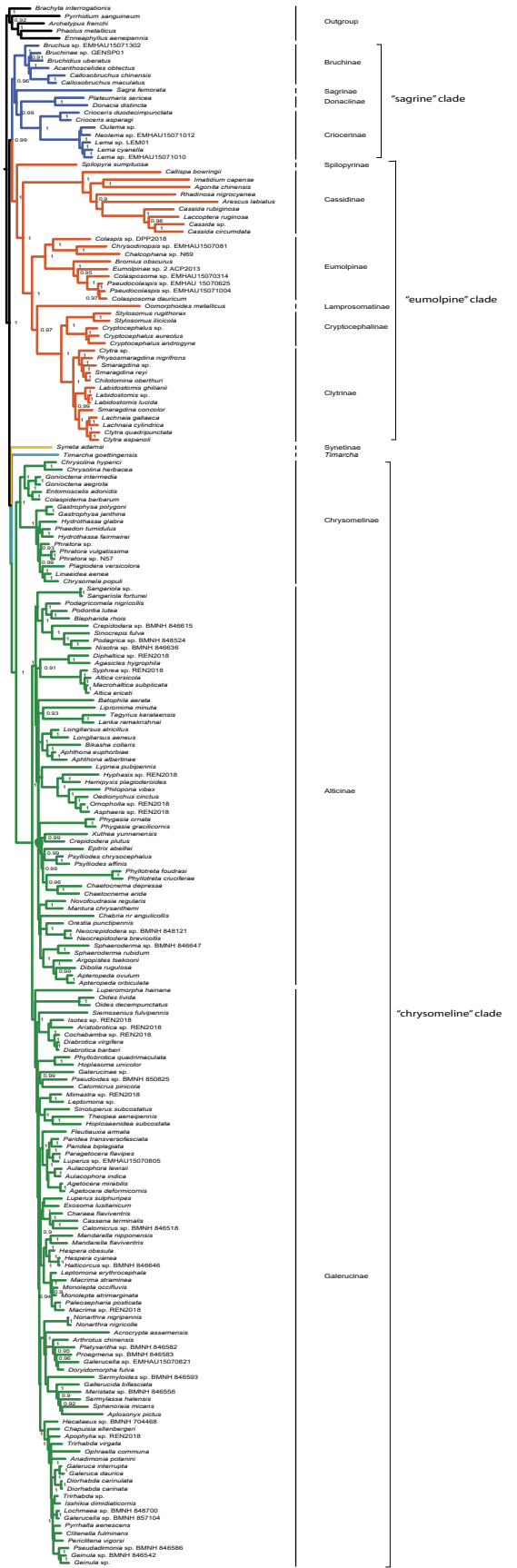

Supplement: S6 Fig — (PDF) [file pone.0258587.s006.pdf]

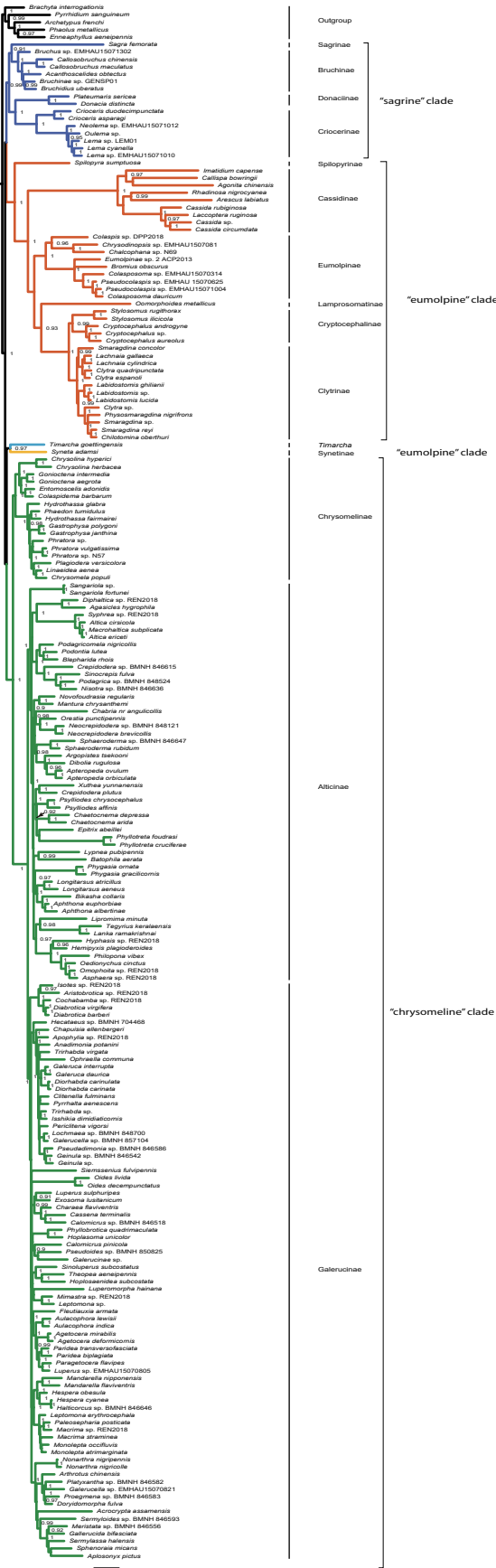

Supplement: S7 Fig — (PDF) [file pone.0258587.s007.pdf]

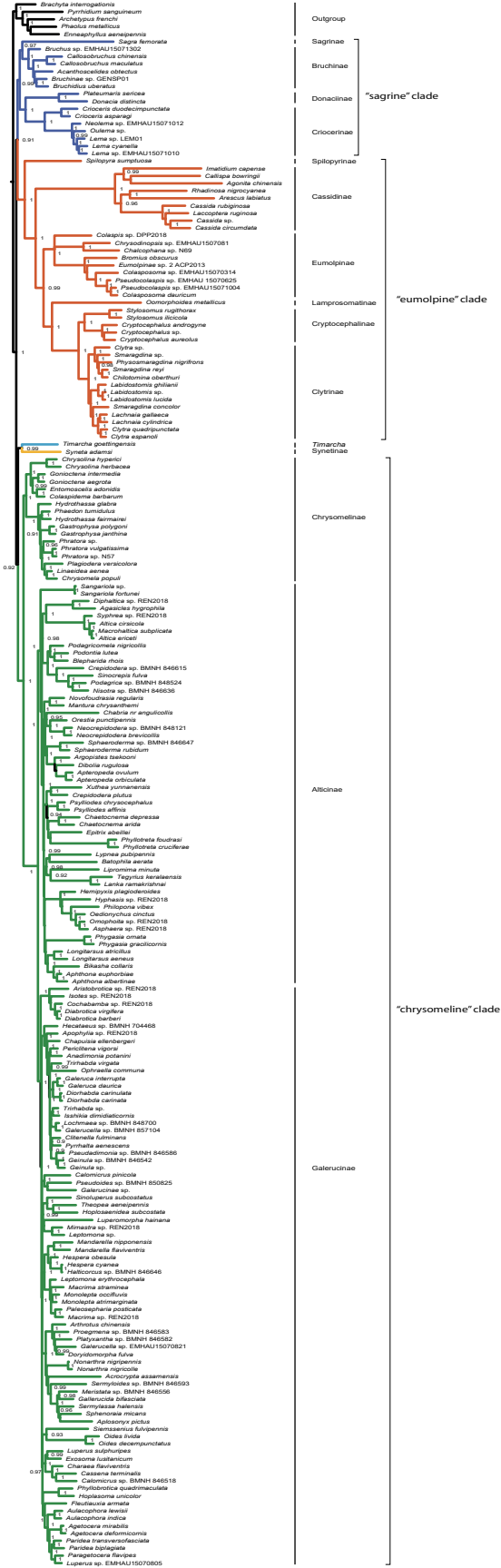

Supplement: S8 Fig — (PDF) [file pone.0258587.s008.pdf]

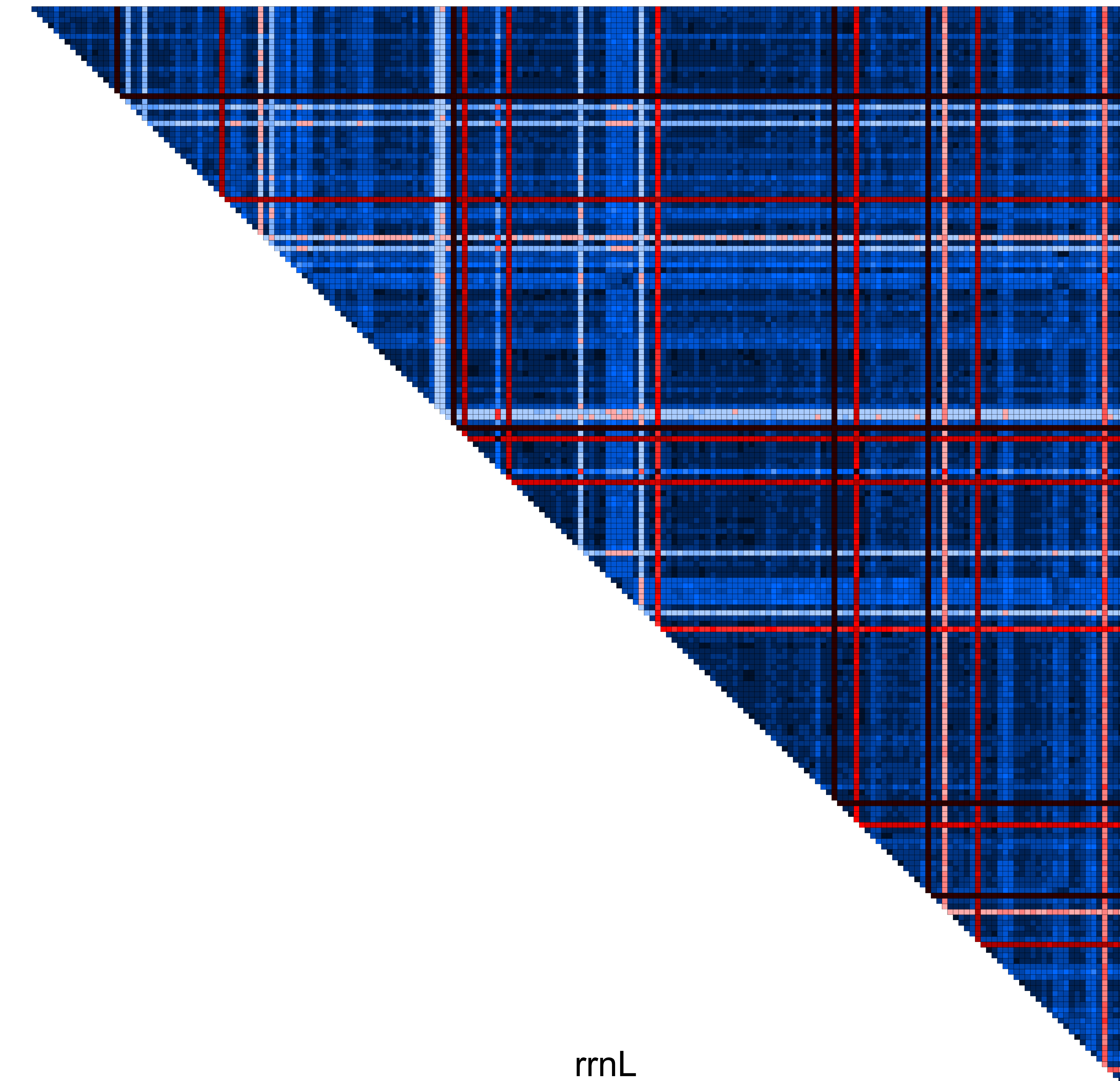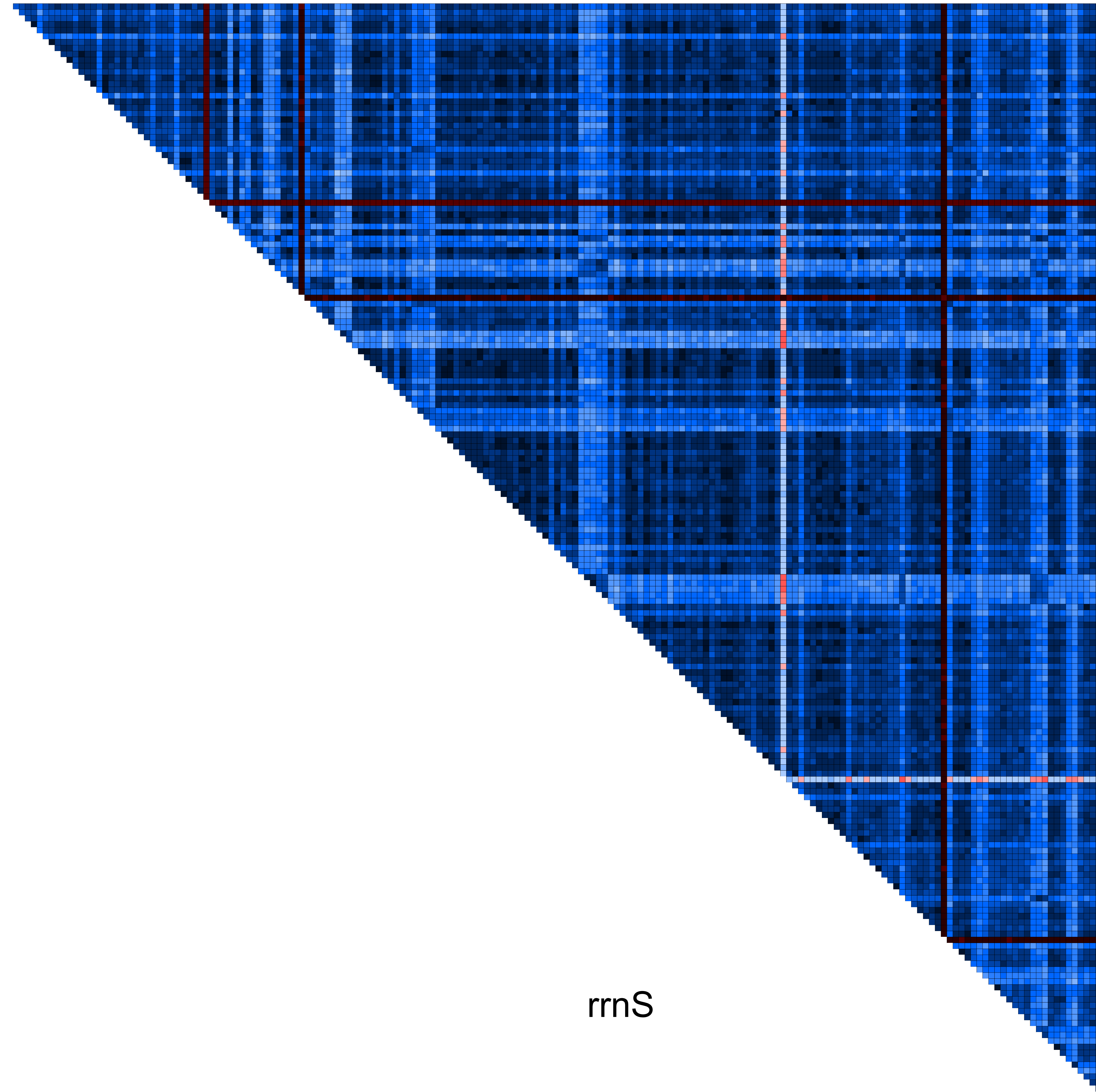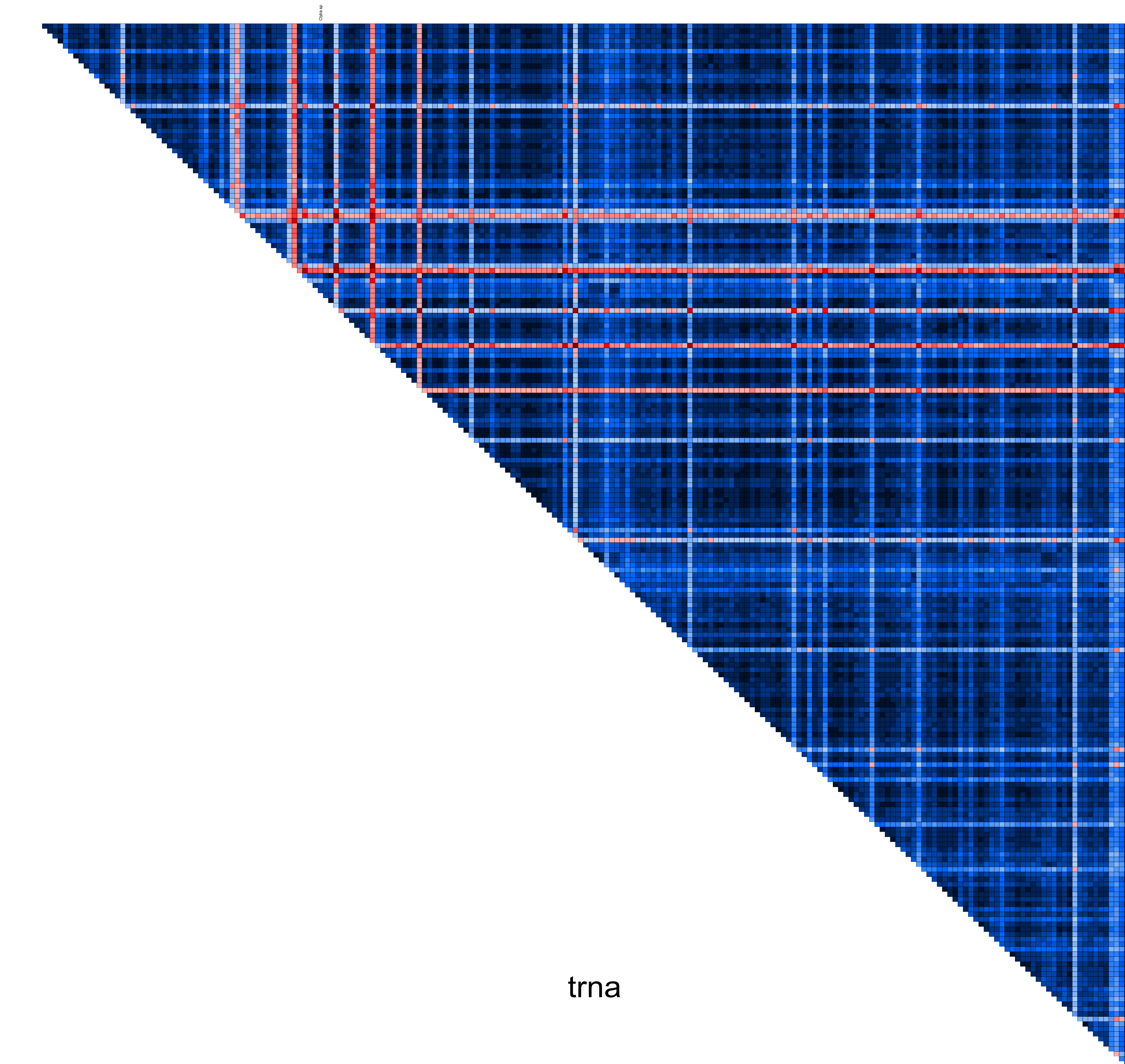

Supplement: S9 Fig — (PDF) [file pone.0258587.s009.pdf]
