# Supplementary material for: Higher-level phylogeny of Chrysomelidae based on expanded sampling of mitogenomes
Source: PLoS One. 2022 Jan 21;17(1):e0258587. doi: 10.1371/journal.pone.0258587 (PMC8782406; doi:10.1371/journal.pone.0258587)

PCGaa_Dayhoff PhyloBayes

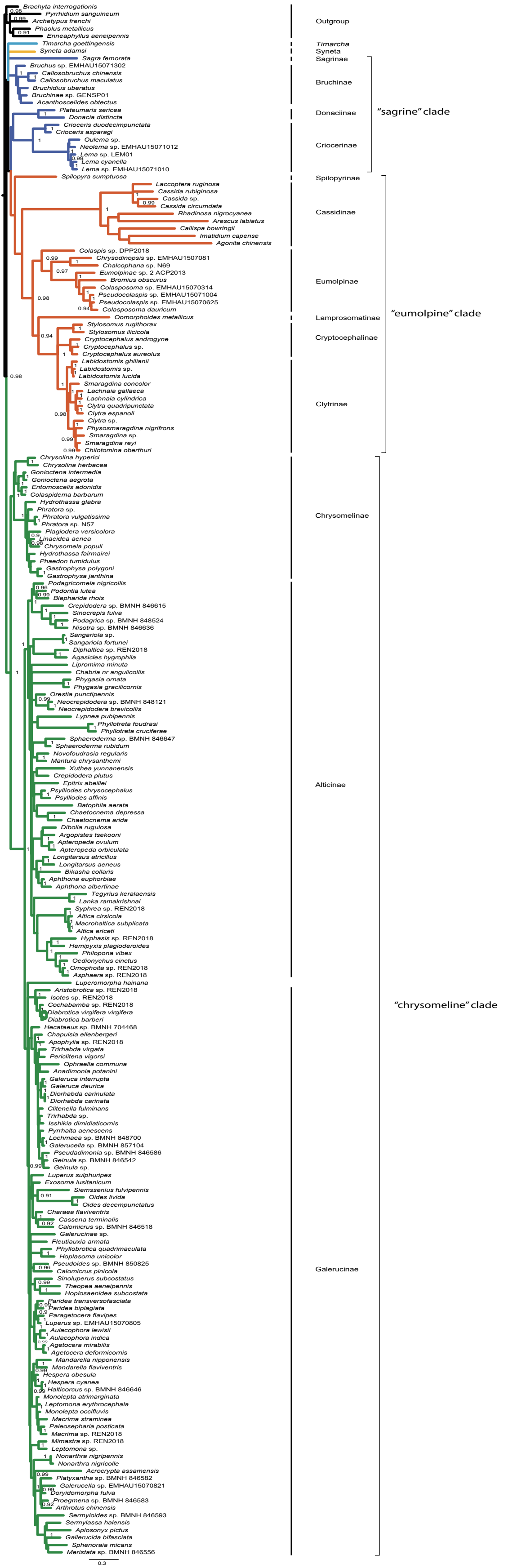

Supplement: S5 Fig — (PDF) [file pone.0258587.s005.pdf]
